# A Smartphone App-Based Lifestyle Change Program for Prediabetes (D'LITE Study) in a Multiethnic Asian Population: A Randomized Controlled Trial

**DOI:** 10.3389/fnut.2021.780567

**Published:** 2022-01-24

**Authors:** Su Lin Lim, Kai Wen Ong, Jolyn Johal, Chad Yixian Han, Qai Ven Yap, Yiong Huak Chan, Zhi Peng Zhang, Cheryl Christine Chandra, Anandan Gerard Thiagarajah, Chin Meng Khoo

**Affiliations:** ^1^Department of Dietetics, National University Hospital, Singapore, Singapore; ^2^Biostatistics Unit, Yong Loo Lin School of Medicine, National University Singapore, Singapore, Singapore; ^3^Department of Family Medicine, National University Polyclinics, Singapore, Singapore; ^4^Division of Endocrinology, National University Hospital, Singapore, Singapore; ^5^Yong Loo Lin School of Medicine, National University of Singapore, Singapore, Singapore

**Keywords:** weight loss, prediabetes, diabetes prevention, obesity, mobile health (mHealth), lifestyle and behavior, intervention - behavioral

## Abstract

**Introduction:**

Landmark studies have established that lifestyle interventions focused on weight loss, diet modification and physical activity can prevent diabetes progression. However, the effectiveness of mobile health application among Asians with prediabetes remains largely unexamined. We aimed to assess whether a smartphone app-based lifestyle intervention program would lead to weight loss, normoglycemia and improved metabolic indices in a multiethnic Asian population with prediabetes.

**Research Design and Methods:**

This multicentre prediabetes RCT is part of the Diabetes Lifestyle Intervention using Technology Empowerment (D'LITE) trial. Adults (*n* = 148) with prediabetes and BMI ≥ 23 kg/m^2^ were randomly allocated either to the intervention group (*n* = 72) empowered by self-monitoring features of the Nutritionist Buddy Diabetes app with in-app dietitian coaching for 6 months, or the control group (*n* = 76) receiving standard diet counseling at baseline. Primary outcome was defined as change in body weight at 6 months, while secondary outcomes included glycemic control and other metabolic indices analyzed using Generalized Linear Mixed Model analysis with intention-to-treat approach.

**Results:**

Intervention group achieved a significantly greater weight loss of 4.2 vs. 1.3 kg [mean difference of −3.1 kg (95% CI −4.5 to −1.7), *p* < 0.001], and a 4.3-fold increased likelihood of achieving ≥ 5% weight loss, as compared to the control group at 6 months. The likelihood of achieving normoglycemia (defined as HbA_1c_ < 5.7%) was 2.1 times higher in intervention group than in the control group (*p* < 0.018). Changes to blood pressure, total and LDL cholesterol were not statistically significant.

**Conclusion:**

An app-based lifestyle program led to clinically significant weight loss and improved glycemia, and can potentially augment current standard care in the prevention of diabetes among an Asian multiethnic population.

**Clinical Trial Registration:**

anzctr.org.au, identifier: ACTRN12617001112358.

## Introduction

Prediabetes is an intermediate hyperglycemic state and part of the metabolic syndrome that increases the risk of diabetes and cardiovascular diseases (1). International Diabetes Federation reported that globally, an estimated 374 million individuals have prediabetes (2). In Singapore, about 14% of the population have been diagnosed with prediabetes (3). In the absence of active intervention, the progression rate of prediabetes to diabetes has been reported to be 6.8% (4). With the anticipated rise in the prevalence of obesity from 4.3% in 1990 to 15.9% in 2050, the lifetime risk of type 2 diabetes in Singapore is forecasted to reach one in two by 2050 (5). It is well-established that Asian populations have a higher risk of developing diabetes at a lower body mass index (BMI) due to the higher predisposition to storing visceral fat than Caucasians (6). This amplifies the public health exigency for upstream preventive measures, particularly in Asian countries.

The US Diabetes Prevention Program (DPP) Study has demonstrated a 58% reduction in diabetes incidence after 3 years of intensive lifestyle intervention among people with impaired glucose tolerance (IGT) (7). Individuals with IGT have increased cardiovascular risk and have eight times higher risk of progressing to diabetes, as compared to normoglycemic individuals (8). Results from the Da Qing and other landmark preventive studies have consistently demonstrated that the trajectory toward type 2 diabetes can be altered by diet and lifestyle modification, with the attainment of weight loss, which is a dominant predictor of reduced prediabetes incidence (9–11). There is a need to translate these trials into community interventions to reduce the disease burden of diabetes and its associated microvascular and macrovascular complications.

Though lifestyle intervention conclusively yields positive outcomes in diabetes prevention, its widespread implementation is impeded due to several limitations. In-person coaching is resource-intensive, time-consuming, costly and requires substantial commuting time, thus limiting the scalability and outreach to high-risk populations (12). Among the screen-and-treat programs studied, only 27% of the high-risk population completed the intervention (13). Low uptake, high attrition and withdrawal rates limit the potential efficacy of these interventions in real-world settings (12–14).

As COVID-19 pandemic continues unabated, there has been a strong push for using mobile health (mHealth) intervention, in place of the traditional in-person modality in health care delivery. Mobile health adoption is set to rise with increasing smartphone ownership as well (15). This opens the possibility of delivering behavioral modification in lifestyle interventions through mobile apps to engage and empower individuals in preventing and managing chronic diseases, as proven in other populations (16–19). This study aimed to examine the effectiveness of an app-based lifestyle intervention with remote dietitian coaching in weight and metabolic management among overweight or obese Asians with prediabetes.

## Materials and Methods

### Study Design and Eligibility

The Diabetes Lifestyle Intervention using Technology Empowerment (D'LITE) trial is a multicenter study comprising of two concurrent parallel RCTs on diabetes and prediabetes. The paper on the diabetes cohort has been published (20) and this paper will focus on the prediabetes group (study protocol in Supplementary Material 1). The study was approved by the National Healthcare Group Domain Specific Review Board in Singapore (2017/00397) and conducted in accordance with the Declaration of Helsinki. Participants were recruited between October 2017 to September 2019 from government polyclinics, general practitioner clinics, health screening facilities and hospital outpatient clinics.

Eligible participants included those aged 21–75 years, diagnosed with prediabetes, with a BMI of 23.0 kg/m^2^ or more, who owned a smartphone and provided written informed consent. Prediabetes was defined as impaired fasting glucose of 6.1–6.9 mmol/L or IGT with 2-h plasma glucose of 7.8–11.0 mmol/L after a 75-g oral glucose tolerance test (21). Exclusion criteria included diagnosis of type 1 or type 2 diabetes, heart failure, advanced kidney disease, depression, severe cognitive deficits, untreated hypothyroidism, pregnancy, untreated anemia, known thalassemia or other blood disorders. This study was prospectively registered with the Australian New Zealand Clinical Trials Registry (ACTRN12617001112358).

### Randomization

Participants were randomized to either intervention or control group using stratified randomization by gender, age (<50 or ≥50 years old) and BMI (<27.5 or ≥27.5 kg/m^2^) after screening. Within each stratified envelopes, an equal proportion of intervention and control group assignments were prepared in advance by a third party not involved in the study and blinded to the study objectives. Eligible participants were block randomized using sealed opaque envelopes in an allocation ratio of 1:1, and aligned with the Consolidated Standards of Reporting Trials (CONSORT-eHEALTH) statement (22). Blinding of participants and investigators was not possible due to the nature of the intervention.

### Treatment

At baseline visit, both control and intervention groups received standard face-to-face dietary advice based on healthy food plate meal-planning principles (23) by a research dietitian. They were all provided with a digital weighing scale (Omron HN-289, Japan) for self-monitoring of their body weight. All participants were also encouraged to engage in 150 min per week of moderate intensity physical activity (24).

Additionally, participants in the intervention group were introduced to the Nutritionist Buddy Diabetes (nBuddy Diabetes) mobile app during the baseline visit. They were required to download the nBuddy Diabetes app and educated to self-monitor their weight, diet, physical activity, and blood glucose levels for 6 months. The nBuddy Diabetes app is designed with an in-built algorithm that incorporates behavioral strategies to empower individuals through prompts and cues. These behavioral strategies include goal-setting, stimulus control, problem solving, self-monitoring, cognitive restructuring and motivational interviewing. The app's automated response system evaluates the suitability of food choices and provides instantaneous feedback to generate a list of healthier and culturally appropriate food alternatives.

The app provided an automated individualized calorie limit which was computed based on body weight, gender, age and activity level. The total daily carbohydrate intake was restricted to 40% of total daily calories. Participants were encouraged to log their meals via the app, with the goal of keeping within the pre-set calorie and carbohydrate limits. As part of the in-app features, self-monitoring of step count using the phone pedometer and physical activity steps conversion function, allowed participants to track their daily step counts. The app automatically set a gradual increase in step count goal starting from 3,000 in the first week to 10,000 steps per day by the third week of the program.

Self-monitoring of weight loss progression and blood glucose level is enabled via the weight and blood glucose logging functions. Participants in the intervention group were advised to monitor and log their weight in the app twice weekly. A glucometer (FreeStyle Optium Neo, United Kingdom) for weekly blood glucose monitoring was also provided. In-app educational videos on weight management, diabetes prevention, healthy meal planning, carbohydrate foods in relation to glycemic response, behavioral strategies, and physical activity were uploaded for each participant on a weekly basis in the first 12 weeks.

For the study period, individualized health coaching was provided based on the participants' app input, which is visible to the research dietitians via the app's dashboard. Through virtual interactions via the app's chat function, the research dietitians facilitated behavioral change (25, 26) by reviewing participants' food intake, step count, weight and glycemic levels regularly, providing real-time feedback and utilizing motivational interviewing skills to guide participants to overcome barriers to change. We had previously reported that the nBuddy app with dietitian's remote coaching resulted in a significant weight loss among patients with type 2 diabetes (20) and non-alcoholic fatty liver disease (25), as well as improvement in glycemic control among patients with type 2 diabetes (20).

### Outcome Evaluation

The primary outcome was mean weight loss from baseline at 6 months post-intervention, with the aim of achieving clinically meaningful weight loss of >5% (27). Body weight after an overnight fast was measured in the clinic by research staff using a standard digital weighing scale (Omron HN-289, Japan), with participants lightly clothed and without shoes. Height was measured without shoes to the nearest centimeter for the calculation of BMI.

Secondary outcomes included mean changes in HbA_1c_, fasting blood glucose (FBG), blood pressure, serum lipids, creatinine, dietary intake, and physical activity. The percentage of participants who achieved normoglycemia in each randomized group was determined. Normoglycemia is defined as HbA_1c_ <5.7% based on American Diabetes Association guidelines (21). Venous blood samples were obtained after 8–12 h of overnight fasting and processed at CAP accredited laboratories (National University Hospital Department of Laboratory Medicine or National Healthcare Group Diagnostics). Plasma glucose was determined by the hexokinase method using photometric assay, and high performance liquid chromatography was used to measure HbA_1c_. Serum lipids and creatinine were measured using enzymatic colorimetric assay.

Blood pressure was measured with participant in a seated position, using an automatic blood pressure monitor (Omron Healthcare) at baseline, and repeated at 3 and 6 months. Participants' physical activity levels in minutes per week were collected using self-reported questionnaires at baseline, 3 and 6 months. Dietary intake was collected using a 2-day food diary at the baseline, 3- and 6-month visits and analyzed using the nBuddy dashboard's nutrient analysis platform, which consists of more than 14,000 food items and incorporates the Singapore Energy & Nutrient Composition of Food, Malaysian Food Composition and USDA food databases, as well as nutritional information from food packaging, and nutrient analysis of recipes. Overall app utilization is defined as the number of days participants actively utilized one or more features of the app over the intervention period.

### Statistical Analysis

The sample size was calculated based on the assumption of at least a moderate Cohen effect size of 0.5 for the difference in weight loss between groups at 6 months. With 90% power at 5% level of significance (two-sided) and attrition rate of 10%, a total of 190 participants (95 per group) was required. All analyses were based on the intention-to-treat approach, and performed using SPSS for Windows version 25.0 (SPSS Inc, Chicago, IL, USA). Markov chain Monte-Carlo multiple imputations method was used to derive missing data points, with predictive mean matching using primary outcome, secondary outcomes, randomization group and demographics to determine each missing value. Continuous variables were presented as mean with standard deviation, while categorical variables were presented as frequencies and percentages. Parametric tests were used where normality and homogeneity assumptions were satisfied, while Mann Whitney U tests were performed when a departure from normal distribution was observed. For categorical variables, the Chi-square or Fisher's exact test was used.

To account for the clustering effect of recruitment sources as a random factor, Generalized Linear Mixed Model analysis was performed on the change from baseline of each of the numerical outcomes. Generalized Poisson Mixed Model was performed for binary outcomes of ≥5% weight loss and HbA_1c_ < 5.7%, adjusting for demographic and relevant covariates, with relative risks presented. Comparison of changes from baseline was performed using paired Student's *t*-test. Statistical significance was set at a two-tailed *P* < 0.05. Between-group Cohen *d* effect sizes were calculated. Type I error for multiple comparisons was adjusted using Benjamini-Hochberg procedure with false discovery rate of 0·2.

## Results

### Participants

**Figure 1:**
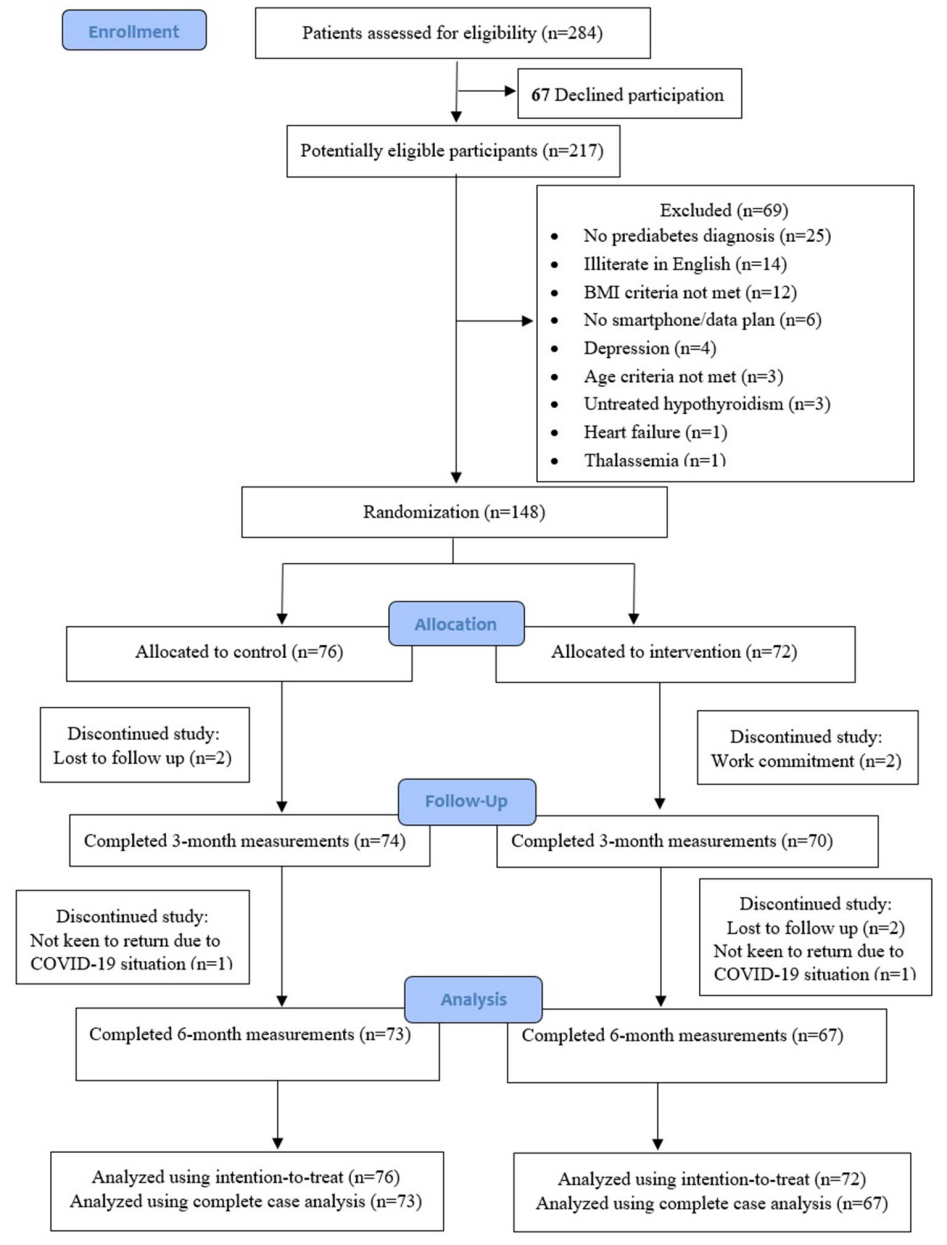
Flowchart of participants screening, recruitment, and randomization.

Figure 1 describes the trial enrollment. Of the 284 potential individuals who were referred and screened for study eligibility, 67 of them declined participation, and another 69 individuals were assessed to be ineligible. A total of 148 (52%) participants were enrolled and randomized to either the intervention group (*n* = 72) or control group (*n* = 76). Five participants from the intervention and three participants from the control group dropped out from the study during the 6-month study period. Complete outcome data were available for 95.9 and 93.2% of participants at 3 and 6 months, respectively.

Participants' baseline characteristics of the intervention and control groups were similar, except for the slightly older age of the control group, compared to the intervention group (Table 1). The mean age of all participants, of whom 60% were male, was 53.1 years (SD 9.3), and their mean BMI was 29.8 kg/m^2^ (SD 4.1). The mean duration of prediabetes was 2.2 years (SD 2.5). None of the participants was prescribed diabetes medication. The proportion of participants with hypertension was 81% and with hyperlipidemia was 81%. The mean total daily calories intake was 1,836 kcal with carbohydrates comprising 48%, protein 17% and fat 35% of total daily calories intake, and mean daily fiber intake was 17 g.

**Table 1:** Baseline characteristics of study participants.

**Variable**	**Control (*n* = 76)**	**Intervention (*n* = 72)**	**Between- group *P*-value**
**Gender**, ***n*** **(%)**
Male	46 (60.5%)	43 (59.7%)	0.920
Female	30 (39.5%)	29 (40.3%)	
**Ethnicity**, ***n*** **(%)**
Chinese	50 (65.8%)	57 (79.2%)	0.080
Malay	16 (21.1%)	7 (9.7%)	
Indian	5 (6.6%)	7 (9.7%)	
Others	5 (6.6%)	1 (1.4%)	
**Age (years)**
Mean	54.3 (9.9)	51.9 (8.7)	0.048
**Weight (kg)**	81.3 (12.5)	82.7 (15.2)	0.659
**BMI (kg/m** ^**2**^ **)**	29.8 (3.9)	29.8 (4.2)	0.816
**Glycemic & Metabolic Control**
HbA_1c_ (%)	6.06 (0.50)	5.94 (0.48)	0.132
Fasting blood glucose (mmol/L)	6.24 (0.79)	6.22 (0.85)	0.705
Systolic blood pressure (mmHg)	135.6 (16.5)	137.3 (18.1)	0.231
Diastolic blood pressure (mmHg)	82.2 (10.5)	83.0 (11.7)	0.655
Total cholesterol (mmol/L)	4.92 (1.03)	5.16 (0.96)	0.135
LDL cholesterol (mmol/L)	2.84 (0.92)	3.13 (0.85)	0.051
HDL cholesterol (mmol/L)	1.32 (0.29)	1.28 (0.25)	0.483
Triglycerides (mmol/L)	1.76 (1.33)	1.70 (0.83)	0.524
Creatinine (umol/L)	80.7 (17.2)	76.9 (17.0)	0.182
Years of prediabetes (years)	2.4 (2.6)	1.9 (2.3)	0.065
**Co-morbidity**, ***n*** **(%)**
Hypertension	63 (82.9%)	57 (79.2%)	0.889
Hyperlipidemia	62 (81.6%)	58 (80.6%)	0.874
Others	9 (11.8%)	8 (11.1%)	0.889
**Nutrient intake**
Calorie, kcal/d	1,868.4 (572.3)	1,804.5 (432.6)	0.937
Carbohydrate, g/d	223.0 (78.6)	213.8 (62.6)	0.899
Sugar, g/d	67.1 (45.1)	62.1 (32.9)	0.818
Protein, g/d	78.5 (27.7)	76.6 (21.7)	0.992
Total fat, g/d	72.8 (25.1)	71.6 (22.0)	0.749
Saturated fat, g/d	29.4 (11.1)	27.5 (8.7)	0.268
Fiber, g/d	17.0 (6.7)	16.8 (5.7)	0.994
**Physical activity, minutes/week**	89.2 (103.0)	102.0 (141.5)	0.748

*Data expressed as mean (SD) for continuous variables or n (%) for categorical variables*.

*BMI, Body Mass Index; HbA_1c_, Hemoglobin A_1c_; LDL, Low-Density Lipoprotein; HDL, High-Density Lipoprotein*.

### Weight Loss

Table 2 describes the changes in primary and secondary outcomes in the intervention and the control groups. At 6 months, there was significantly greater weight loss in the intervention group than the control group [−4.2 kg (4.5) vs. −1.3 kg (3.9); *P* < 0.001]. The mean percentage weight loss achieved among the intervention and control groups were 5.2 and 1.5%, respectively (*P* < 0.001), with a moderate Cohen *d* effect size of 0.76. Sensitivity analyses using complete case analysis resulted in similar findings (Supplementary Tables 1–3). The intervention group had a 4.3 times higher likelihood of achieving ≥ 5% weight loss at 6 months than the control group after adjustment for gender, ethnicity and age (95% CI 2.0–9.2, *P* < 0.001) (Table 3).

**Table 2:** Primary and secondary outcomes at 3 and 6 months post-enrollment.

**Outcomes**	***n***	**Mean change from baseline**		**Between-group differences**		
		**Control (*n* = 76)**	**Intervention (*n* = 72)**	**Mean difference (95% CI)**	***P-*value^a^**	**Cohen *d***
**Δ** **Weight, kg**						
3-month	148	−1.0 (3.0)^*^	−3.6 (3.6)^*^	−2.7 (−3.9 to −1.5)	<0.001^**^	0.78
6-month	148	−1.3 (3.9)^*^	−4.2 (4.5)^*^	−3.1 (−4.5 to −1.7)	<0.001^**^	0.69
**Δ** **Weight, %**						
3-month	148	−1.1 (3·2)	−4·4 (4·3)	−3.4 (−4.7 to −2.1)	<0.001^**^	0.87
6-month	148	−1.5 (4.2)	−5.2 (5.4)	−3.9 (−5.5 to −2.3)	<0.001^**^	0.76
**Δ** **BMI, kg/m**^2^						
3-month	148	−0.4 (1.2)^*^	−1.2 (1.4)^*^	−0.9 (−1.4 to −0.4)	0.001^**^	0.61
6-month	148	−0.5 (1.5)^*^	−1.5 (1.7)^*^	−1.1 (−1.6 to −0.5)	<0.001^**^	0.62
**Δ** **HbA**_**1c**_**, %**						
3-month	148	−0.07 (0.31)	−0.16 (0.27)^*^	−0.10 (−0.20 to 0)	0.049^**^	0.31
6-month	148	−0.06 (0.26)^*^	−0.22 (0.33)^*^	−0.19 (−0.28 to −0.09)	<0.001^**^	0.54
**Δ** **Fasting blood glucose, mmol/L**						
3-month	148	−0.09 (0.67)	−0.25 (0.82)^*^	−0.23 (−0.43 to −0.03)	0.024^**^	0.21
6-month	148	0.01 (0.74)	−0.37 (0.88)^*^	−0.44 (−0.68 to −0.20)	<0.001^**^	0.47
**Δ** **Systolic blood pressure, mmHg**		***n*** **=** **63**	***n*** **=** **57**			
3-month	120	−3.8 (15.8)	−5.1 (13.3)^*^	0.8 (−4.0 to 5.6)	0.749	0.09
6-month	120	−2.5 (17)	−6.1 (14.2)^*^	−1·2 (−5.9 to 3.6)	0.622	0.23
**Δ** **Diastolic blood pressure, mmHg**						
3-month	120	0.5 (9.7)	−1.7 (8.8)	−2.1 (−5.0 to 0.7)	0.147	0.24
6-month	120	−1.9 (9.6)	−3.2 (10.4)^*^	−1.0 (−4.2 to 2.2)	0.541	0.13
**Δ** **Total cholesterol**, **mmol/L**		*n* = 62	*n* = 58			
3-month	120	−0.19 (0.86)	−0.25 (0.85)^*^	0.08 (−0.21 to 0.36)	0.598	0.07
6-month	120	−0.17 (0.99)	−0.29 (0.89)^*^	0.01 (−0.28 to 0.30)	0.945	0.13
**Δ** **LDL cholesterol, mmol/L**						
3-month	120	−0.13 (0.77)	−0.17 (0.78)	0.12 (−0.13 to 0.38)	0.341	0.05
6-month	120	−0.13 (0.95)	−0.31 (0.84)^*^	0·01 (−0.27 to 0.28)	0.959	0.20
**Δ** **HDL cholesterol, mmol/L**						
3-month	120	0.02 (0.18)	0.03 (0.14)	−0.01 (−0.07 to 0.05)	0.686	0.06
6-month	120	0 (0.23)	0.09 (0.22)^*^	0.08 (0 to 0.15)	0.046^**^	0.40
**Δ** **Triglycerides, mmol/L**						
3-month	120	−0.28 (1.26)	−0.26 (0.75)^*^	−0.12 (−0.37 to 0.13)	0.349	0.02
6-month	120	−0·11 (1·58)	−0·19 (0·77)	−0.26 (−0.64 to 0.11)	0.165	0.06
**Δ** **Creatinine, umol/L**						
3-month	148	−2.2 (8.1)^*^	0.4 (7.6)	1·8 (−0.7 to 4.3)	0.149	0.33
6-month	148	−2.2 (7.3)^*^	1.0 (7.0)	3·0 (0.6 to 5.3)	0.015^**^	0.45
**Δ** **Calorie, kcal/d**						
3-month	148	−226.6 (658.7)^*^	−500.3 (449.0)^*^	−326.5 (−458.1 to −194.9)	<0.001^**^	0.49
6-month	148	−129.4 (612.5)	−496.5 (461.5)^*^	−397·1 (−530.4 to −263.8)	<0.001^**^	0.68
**Δ** **Carbohydrate, g/d**						
3-month	148	−30.0 (82.0)^*^	−67.0 (65.1)^*^	−42.2 (−58.9 to −25.6)	<0.001^**^	0.50
6-month	148	−17.6 (76.4)^*^	−67.2 (61.1)^*^	−53.5 (−70.1 to −36.8)	<0.001^**^	0.72
**Δ** **Sugar, g/d**						
3-month	148	−14.5 (41.4)^*^	−28.2 (35.2)^*^	−16.7 (−24.2 to −9.3)	<0.001^**^	0.36
6-month	148	−12.1 (42.5)^*^	−28.1 (35.7)^*^	−19.0 (−26.7 to −11.3)	<0.001^**^	0.41
**Δ** **Protein, g/d**
3-month	148	−8.2 (32.3)^*^	−11.0 (23.9)^*^	−5.1 (−11.7 to 1.5)	0.132	0.10
6-month	148	−2.5 (37.2)	−11.8 (25.9)^*^	−10.8 (−18.7 to −2.9)	0.007^**^	0.29
**Δ** **Total fat, g/d**						
3-month	148	−8.5 (31.4)^*^	−22.7 (24.0)^*^	−15.4 (−22.5 to −8.4)	<0.001^**^	0.51
6-month	148	−5.0 (31.8)	−19.8 (25.7)^*^	−15.4 (−22.8 to −8.0)	<0.001^**^	0.51
**Δ** **Saturated fat, g/d**						
3-month	148	−4.2 (14.5)^*^	−10.4 (10.0)^*^	−7.6 (−10.7 to −4.4)	<0.001^**^	0.50
6-month	148	−2.4 (13.2)	−8.2 (10.9)^*^	−7.1 (−10.3 to −3.9)	<0.001^**^	0.48
**Δ** **Fiber, g/d**						
3-month	148	−1.8 (7.5)^*^	−2.3 (5.7)^*^	−0·5 (−2.2 to 1.1)	0.526	0.08
6-month	148	−0.4 (7.5)	−1.4 (7.0)	−0·8 (−2.7 to 1.1)	0.398	0.14
**Δ** **Physical activity, minutes/week**						
3-month	148	10.1 (105.0)	43.1 (127.3)^*^	42.5 (6.9 to 78.1)	0.019^**^	0.28
6-month	148	11.2 (123.4)	44·2 (144.8)^*^	33.1 (−4.5 to 70.7)	0.084	0.25

a *Adjusted for gender, race, age and baseline value of the outcomes*.

* *Significant within-group changes with P < 0.05 after Benjamini-Hochberg correction with false discovery rate at 0·2 and n = 76*.

** *Significant adjusted P-values after Benjamini-Hochberg correction with false discovery rate at 0·2 and n = 40*.

**Table 3:** Proportion of participants with ≥ 5% weight loss.

	**Control (76) *n* (%)**	**Intervention (72) *n* (%)**	**Unadjusted RR (95% CI)**	***P*-value**	**Adjusted RR (95% CI)^a^**	***P*-value^a^**
3-month	6 (7·9%)	31 (43·1%)	5·8 (2·3 to 14·5)	<0·001	6·6 (2·6 to 16·8)	<0·001
6-month	9 (11·8%)	33 (45·8%)	3·9 (1·8 to 8·2)	<0·001	4·3 (2·0 to 9·2)	<0·001

a *Adjusted for gender, race and age*.

*RR, Relative risk; the control group is the reference*.

### Glycemic and Metabolic Control

Normoglycemia, defined as HbA_1c_ < 5.7%, was 2.1 times more likely to be achieved among the intervention group, as compared to control group (Table 4). At 6 months, the proportion of participants with normoglycemia doubled to 44.4% in the intervention group, compared to 23.7% in control participants. The mean reduction in HbA_1c_ [−0.22% (0.33) vs. −0.06% (0.26)] and FBG [−0.37 mmol/L (0.88) vs. 0.01 mmol/L (0.74)] were significantly greater (*P* <0.001) in the intervention, as compared to control group at 6 months.

**Table 4:** Proportion of participants who achieved normoglycemia (HbA1c <5.7%) at 6 months.

		**Unadjusted**		**Adjusted** ^a^	
**Control (76) *n* (%)**	**Intervention (72) *n* (%)**	**RR (95% CI)**	***P*-value**	**RR (95% CI)**	***P*-value**
18 (23.7%)	32 (44.4%)	1.9 (1.1–3.4)	0.035	2.1 (1.1–3.9)	0.018

a *Adjusted for gender, race and age*.

*RR, Relative risk; the control group is the reference*.

There were significant within-group improvements in systolic and diastolic blood pressure, total cholesterol and Low-Density Lipoprotein (LDL) cholesterol in the intervention group, but they did not reach statistical significance, when compared to the control group. The between-group difference reached statistical significance for High-Density Lipoprotein (HDL) cholesterol (*P* = 0.046), favoring the intervention group. Although between-group difference in serum creatinine concentration was statistically significant, the difference was not clinically relevant.

### Dietary Intake and Physical Activity

At 6 months, the intervention group had significantly greater reductions in total daily calories, carbohydrate, total fat, saturated fat and sugar intake, compared to the control group (*P* < 0.001) (Table 2). Compared to baseline level, the intervention group had a significantly higher physical activity at 6 months; however, the between-group difference did not reach statistical significance (*P* = 0.084).

### App Utilization

The median (interquartile range) of the overall app utilization in the intervention group was 97.8% (85.3–100%) during the first 3-month and 91.7% (51.1–100%) during 4 to 6-month of intervention period. The average two-way dietitian-to-participant interactions via the app's chat function were 3 days per week in the first 3 months, and 2 days per week in the subsequent 3 months.

## Discussion

The D'LITE prediabetes study showed that a smartphone app-based lifestyle program with in-app dietitian coaching resulted in significantly greater weight loss, when compared to the control group, among a multiethnic Asian population with prediabetes. Additionally, participants in the intervention group were more likely to achieve clinically meaningful glycemic improvement, resulting in normoglycemia. These results could translate to potential application in future diabetes prevention program.

Several diabetes prevention trials have reported that weight loss is the main determinant of diabetes prevention (28), and lifestyle intervention using mHealth has the potential to lower diabetes occurrence among the target group (16–18). Systematic review and meta-analysis of diabetes prevention programs had reported mean weight loss ranging from 2.6 to 4.3% with study durations ranging from 3 to 12 months (29, 30). The findings of this study add to the literature that in addition to face-to-face dietary intervention, an app-based diabetes prevention intervention is feasible and yields a comparable weight loss (31).

The US Centers for Disease Control and Prevention (CDC) recommends a clinically meaningful weight loss of 5% for lifestyle programs to prevent diabetes (32). Similar to Block et al. (18), this study showed that 43.1% (31/72) of the participants in the app-based intervention group achieved at least 5% weight loss by 3 months, and 45.8% (33/72) of them achieved the same at 6 months. It does appear that a substantial amount of weight loss was achieved within the first 3 months of high app engagement, with subsequent weight loss maintenance at 6 months. Participants in the app-based lifestyle intervention had a 4.3 times likelihood of achieving the desired weight loss target at 6 months, compared to control group. Our results reaffirm the positive impact of weight loss in retarding the development of diabetes (33), echoing findings of the US DPP study which reported a 16% reduction in the relative risk of diabetes for every kilogram of weight loss (34).

D'LITE study is one of the few RCTs that assess the effectiveness of using a mobile app along with virtual dietitian health coaching in a single platform to facilitate lifestyle modifications for diabetes prevention (35). The app is designed with an automated algorithm which provides real-time feedback with personal health data tracking, along with personalized health coaching via the app's chat function. With the current study results, there is a possibility that we can leverage on a digital diabetes prevention program to scale up diabetes preventive efforts targeting people at risk of diabetes.

It is well known that adherence to a diabetes prevention program is a challenge for many people. Hence, a digital platform may bridge the gap for people to access the program at their own convenience and psychological comfort. Currently, most mobile apps support self-monitoring behaviors such as calorie counting and step tracking but without the synchronous or asynchronous feedback from the health care professional or dietitian (36). In addition to the aforementioned features, the nBuddy Diabetes app provides automated calories and carbohydrate evaluation, culturally appropriate healthier alternatives and individualized real-time health coaching. We believe that these additional features acting in combination could have empowered participants to achieve the desired health goals.

Large RCTs have shown that diet and lifestyle modification is the first-line intervention for prevention of diabetes (16–18). At 6 months, participants in the app-based intervention group had significantly lower total daily energy intake of about 500 kcal, lower total carbohydrate, total fat and saturated fat intake, compared to the control group. Also, compared to the control group, the participants in the intervention group achieved a significantly higher physical activity per week at 3 months. The combination of the above could have contributed to the observed improvements in body weight, BMI, HbA_1c_ and FBG, and if efforts are sustained, would likely result in diabetes prevention. Importantly, we showed that the D'LITE study intervention was able to produce meaningful weight loss and glycemic improvements despite being less intensive and of a shorter duration than the DPP intervention with its in-person health coaching and supervised group exercise sessions (34).

Lifestyle interventions need to be individualized to better facilitate behavioral changes (26), as evidenced by the lack of weight change between intervention and control groups in the absence of health coach or app customization (17). Our study results concur with a systematic review by Joiner et al. (16) which demonstrated that technology combined with online health coaching resulted in greater weight loss compared to fully automated electronic health interventions. Incorporating in-app coaching from dietitians allows some degree of tailoring the intervention to better suit the individuals. The nBuddy Diabetes platform allows access to an extensive local food database and culturally specific food alternatives. It also provides automated prompts on lifestyle messages and timely feedback, thus reducing manpower costs associated with multiple in-person health coaching, improving program accessibility and scalability, while re-enforcing self-empowerment behaviors among users.

We also observed that there was a significant within-group reduction in the lipid profile, systolic, and diastolic blood pressure among participants in the app-based intervention group at 6 months which could be attributed to the significant reduction in body weight and lifestyle changes. Taken together, the app-based intervention improved hyperglycemia, hypertension, and hyperlipidemia, the major three important risk factors of cardiovascular disease.

The strengths of this study include a stratified randomized approach to ensure that the baseline characteristics were balanced between the intervention and control group. We also adopted multisource recruitment for better representation of the general population. There was a low attrition rate and high percentage of complete outcome data at 6 months. Digital health, with the flexible engagement, constant motivation and reduced time commitment and burden associated with attending in-person programs, appears to improve retention of participants. The intention-to-treat analysis ensures that type I error is minimized, allowing for greater generalizability of our results. Lastly, our study is among the few RCTs in Asia which assessed the effectiveness of an integrated mobile app with remote health coaching by dietitians.

This study has several limitations. It included smartphone users who were literate in English, and would have excluded non-English speaking people from minority groups. Further studies to examine the effectiveness of the nBuddy app among users of other languages will be logical, but we opine that this will only enhance the existing findings as this study already had representation from the three major Asian ethnic groups with distinct cultural beliefs and languages. Although the trial was not able to enroll the original sample size due to the lower than anticipated recruitment rate within the stipulated study timeline, it was still able to provide significant results of the treatment effect.

Another limitation lies in the use of a self-administered questionnaire rather than an objective pedometer for determining physical activity level, as questionnaire response bias could influence results. Finally, the scope of this paper is limited to 6 months of intervention. It is not known if the beneficial effect of the app-based intervention would be sustained over a longer period of time. However, such longitudinal data will be published in subsequent papers when the follow-up of study participants at 1 and 2 years is completed and analyzed.

In conclusion, a 6 months app-based lifestyle intervention program significantly reduced body weight and BMI, and improved metabolic profile of individuals with prediabetes from a multiethnic Asian cohort. This study provides evidence that an app-based lifestyle intervention with remote dietitian coaching is feasible and potentially scalable to reach a wider population of people with prediabetes.

## Data Availability Statement

The original contributions presented in the study are included in the article/Supplementary Material, further inquiries can be directed to the corresponding author/s.

## Ethics Statement

The studies involving human participants were reviewed and approved by NHG Domain Specific Review Board. The patients/participants provided their written informed consent to participate in this study.

## Author Contributions

SLL is the Principal Investigator and guarantor of this work and, as such, had full access to all the data in the study and takes responsibility for the integrity of the data and the accuracy of the data analysis. SLL and CMK conceived of the presented idea, developed the theory, and study design. SLL, KWO, JJ, CYH, ZPZ, CCC, and AGT carried out the project. QVY and YHC analyzed the data and provided statistical expertise. KWO, SLL, and CMK wrote the manuscript. All authors were involved in the manuscript review and approved the version submitted for publication.

## Funding

The D'LITE study was funded by the Singapore Ministry of Health's National Medical Research Council under its Health Services Research Grant (NMRC/HSRG/0063/2016) which had no role in the study design, data collection, data analysis, data interpretation or report writing.

## Conflict of Interest

The authors declare that the research was conducted in the absence of any commercial or financial relationships that could be construed as a potential conflict of interest.

## Publisher's Note

All claims expressed in this article are solely those of the authors and do not necessarily represent those of their affiliated organizations, or those of the publisher, the editors and the reviewers. Any product that may be evaluated in this article, or claim that may be made by its manufacturer, is not guaranteed or endorsed by the publisher.
