# Supplementary material for: A Smartphone App-Based Lifestyle Change Program for Prediabetes (D'LITE Study) in a Multiethnic Asian Population: A Randomized Controlled Trial
Source: Front Nutr. 2022 Jan 24;8:780567. doi: 10.3389/fnut.2021.780567 (PMC8819073; doi:10.3389/fnut.2021.780567)
Supplement: Supplementary file 2 [file Data_Sheet_2.docx]

**Supplement 2**

**eTable 1: Primary and secondary outcomes at 3 and 6 months post-enrollment (complete case analysis)**

| Outcomes | n | Mean Change from Baseline | | Between-group differences | | | |
| --- | --- | --- | --- | --- | --- | --- | --- |
|  |  | Control  (n = 76) | Intervention  (n = 72) | Mean difference (95% CI) | *P* value^a^ | Cohen *d* | |
| ∆ Weight, kg |  |  |  |  |  | |  |
| 3-month | 144 | -0·7 (2·0)* | -3·3 (3·0)* | -2·7 (-3·5 – -1·8) | <0·001** | | 1·02 |
| 6-month | 140 | -0·9 (2·6)* | -4·0 (3·8)* | -3·2 (-4·3 – -2·1) | <0·001** | | 0·95 |
| ∆ Weight, % |  |  |  |  |  | |  |
| 3-month | 144 | -0·9 (2·5) | -4·2 (3·7) | -3·4 (-4·5 – -2·3) | <0·001** | | 1·05 |
| 6-month | 140 | -1·2 (3·3) | -5·0 (4·8) | -4·0 (-5·3 – -2·6) | <0·001** | | 0·92 |
| ∆ BMI, kg/m^2^ |  |  |  |  |  | |  |
| 3-month | 144 | -0·3 (0·7)* | -1·2 (1·1)* | -0·9 (-1·3 – -0·6) | <0·001** | | 0·98 |
| 6-month | 140 | -0·4 (1·0)* | -1·4 (1·4)* | -1·1 (-1·5 – -0·7) | <0·001** | | 0·82 |
| ∆ HbA_1c_, % |  |  |  |  |  | |  |
| 3-month | 142 | -0·07 (0·28)* | -0·17 (0·26)* | -0·10 (-0·19 – -0·01) | 0·027** | | 0·37 |
| 6-month | 139 | -0·07 (0·26)* | -0·22 (0·30)* | -0·19 (-0·28 – -0·10) | <0·001** | | 0·53 |
| ∆ Fasting Blood Glucose, mmol/L |  |  |  |  |  | |  |
| 3-month | 142 | -0·07 (0·64) | -0·24 (0·79)* | -0·23 (-0·42 – -0·04) | 0·017** | | 0·24 |
| 6-month | 139 | 0·02 (0·69) | -0·37 (0·84)* | -0·45 (-0·67 – -0·23) | <0·001** | | 0·51 |
| ∆ Systolic blood pressure, mmHg |  | n = 63 | n = 57 |  |  | |  |
| 3-month  6-month | 117  112 | -3·4 (15·6)  -2·0 (16·6) | -5·2 (13·0)*  -5·8 (14·0)* | 0·3 (-4·4 – 4·9)  -1·5 (-6·4 – 3·4) | 0·909  0·549 | | 0·13  0·25 |
| ∆ Diastolic blood pressure, mmHg |  |  |  |  |  | |  |
| 3-month | 117 | 0·3 (9·6) | -1·7 (8·8) | -2·1 (-5·0 – 0·8) | 0·161 | | 0·22 |
| 6-month | 112 | -1·6 (9·2) | -3·2 (10·2)* | -1·5 (-4·5 – 1·6) | 0·353 | | 0·16 |
| ∆ Total cholesterol, mmol/L |  | n = 62 | n = 58 |  |  | |  |
| 3-month | 115 | -0·14 (0·82) | -0·21 (0·80) | 0·06 (-0·22 – 0·34) | 0·663 | | 0·09 |
| 6-month | 111 | -0·14 (0·80) | -0·21 (0·66)* | 0·02 (-0·23 – 0·26) | 0·892 | | 0·10 |
| ∆ LDL cholesterol, mmol/L |  |  |  |  |  | |  |
| 3-month | 113 | -0·09 (0·69) | -0·14 (0·72) | 0·10 (-0·15 – 0·35) | 0·428 | | 0·07 |
| 6-month | 109 | -0·11 (0·71) | -0·23 (0·58)* | 0·01 (-0·21 – 0·22) | 0·962 | | 0·19 |
| ∆ HDL cholesterol, mmol/L |  |  |  |  |  | |  |
| 3-month | 115 | 0·01 (0·18) | 0·03 (0·13) | 0 (-0·06 – 0·06) | 0·989 | | 0·13 |
| 6-month | 111 | -0·02 (0·17) | 0·08 (0·20)* | 0·09 (0·02 – 0·16) | 0·016** | | 0·54 |
| ∆ Triglycerides, mmol/L |  |  |  |  |  | |  |
| 3-month | 115 | -0·27 (1·26) | -0·26 (0·75)* | -0·15 (-0·41 – 0·10) | 0·240 | | 0·01 |
| 6-month | 111 | -0·13 (1·59) | -0·14 (0·54) | -0·27 (-0·65 – 0·11) | 0·159 | | 0·01 |
| ∆ Creatinine, umol/L |  |  |  |  |  | |  |
| 3 month | 142 | -2·1 (8·1)* | 0·4 (7·6) | 1·8 (-0·7 – 4·4) | 0·151 | | 0·32 |
| 6 month | 138 | -2·2 (7·3)* | 1·0 (7·0) | 3·0 (0·6 – 5·4) | 0·016** | | 0·45 |
| ∆ Calorie, kcal/d |  |  |  |  |  | |  |
| 3 month | 142 | -211·4(619·3)* | -490·7 (444·3)* | -343·8 (-473·1- 214·5) | <0·001** | | 0·52 |
| 6 month | 140 | -104·1 (555·1) | -524·9 (436·2)* | -429·7 (-556·6– -302·7) | <0·001** | | 0·84 |
| ∆ Carbohydrate, g/d |  |  |  |  |  | |  |
| 3 month | 142 | -26·0 (71·8)* | -65·9 (64·4)* | -45·1 (-60·7 – -29·5) | <0·001** | | 0·59 |
| 6 month | 140 | -13·4 (65·3) | -69·7 (57·9)* | -57·6 (-73·8 – -41·4) | <0·001** | | 0·91 |
| ∆ Sugar, g/d |  |  |  |  |  | |  |
| 3 month | 142 | -11·4 (31·8)* | -27·6 (34·7)* | -17·4 (-24·4 – -10·5) | <0·001** | | 0·49 |
| 6 month | 140 | -9·7 (35·0)* | -28·7 (35·7)* | -19·3 (-26·9 – -11·7) | <0·001** | | 0·54 |
| ∆ Protein, g/d |  |  |  |  |  | |  |
| 3 month | 142 | -8·7 (31·5)* | -10·4 (22·5)* | -5·1 (-11·8 – 1·5) | 0·131 | | 0·06 |
| 6 month | 140 | -2·3 (34·9) | -11·9 (22·9)* | -11·6 (-18·8 – -4·4) | 0·002** | | 0·33 |
| ∆ Total fat, g/d |  |  |  |  |  | |  |
| 3 month | 142 | -8·2 (30·5)* | -22·3 (24·1)* | -15·9 (-23·0 – -8·7) | <0·001** | | 0·51 |
| 6 month | 140 | -4·2 (30·7) | -21·5 (24·5)* | -17·0 (-23·9 – -10·0) | <0·001** | | 0·62 |
| ∆ Saturated fat, g/d |  |  |  |  |  | |  |
| 3 month | 142 | -4·1 (14·2)* | -10·2 (9·9)* | -7·8 (-10·9 – -4·7) | <0·001** | | 0·50 |
| 6 month | 140 | -2·2 (12·8) | -9·1 (10·4)* | -7·8 (-11·0 – -4·6) | <0·001** | | 0·59 |
| ∆ Fiber, g/d |  |  |  |  |  | |  |
| 3 month | 142 | -1·8 (7·4)* | -2·5 (5·6)* | -0·6 (-2·3 – 1·0) | 0·438 | | 0·11 |
| 6 month | 140 | -0·5 (7·5) | -1·7 (6·4)* | -0·9 (-2·7 – 0·9) | 0·320 | | 0·17 |
| ∆ Physical activity, minutes/week |  |  |  |  |  | |  |
| 3 month | 144 | 6·4 (87·9) | 42·2 (125·8)* | 45·7 (12·1 – 79·3) | 0·008** | | 0·33 |
| 6 month | 140 | 7·7 (112·4) | 40·3 (129·1)* | 33·5 (-3·3 – 70·4) | 0·074 | | 0·27 |

^a^adjusted for gender, race, age and baseline value of the outcome

*Significant within group changes *P* values after Benjamini-Hochberg correction with false discovery rate at 0·2 and n=76.

**Significant adjusted *P* values after Benjamini-Hochberg correction with false discovery rate at 0·2 and n=40.

**eTable 2: Proportion of participants with ≥ 5% weight loss**

|  |  |  | Unadjusted | | Adjusted^e^ | |
| --- | --- | --- | --- | --- | --- | --- |
|  | Control  n (%) | Intervention n (%) | RR (95% CI) | *P* value | RR (95% CI) | *P* value |
| 3-month  6-month | 4 (5·4%)^a^  7 (9·6%)^c^ | 29 (41·4%)^b^  31 (46·3%)^d^ | 7·7 (2·7 – 22·0)  4·8 (2·1 – 11·0) | <0·001  <0·001 | 8·6 (2·9 – 25·2)  5·2 (2·2 – 12·1) | <0·001  <0·001 |

^a^N = 74

^b^N = 70

^c^N = 73

^d^N = 67

^e^adjusted for gender, race and age

RR: Relative risk; the control group is the reference

**eTable 3: Proportion of participants who achieved normoglycemia (HbA1c < 5.7%) at 6 months.**

|  |  | Unadjusted | | Adjusted^c^ | |
| --- | --- | --- | --- | --- | --- |
| Control  n (%)^a^ | Intervention n (%)^b^ | RR (95% CI) | P value | RR (95% CI) | P value |
| 15(20.5%) | 30(45.5%) | 2.2 (1.2- 4.1) | 0.013 | 2.4 (1.3- 4.7) | 0.009 |

^a^N = 73

^b^N = 66

^c^adjusted for gender, race and age

RR: Relative risk; the control group is the reference
